# Where Actions Meet Outcomes: Medial Prefrontal Cortex, Central Thalamus, and the Basal Ganglia

**DOI:** 10.3389/fnbeh.2022.928610

**Published:** 2022-07-05

**Authors:** Robert G. Mair, Miranda J. Francoeur, Erin M. Krell, Brett M. Gibson

**Affiliations:** ^1^Department of Psychology, The University of New Hampshire, Durham, NH, United States; ^2^Neural Engineering and Translation Labs, University of California, San Diego, San Diego, CA, United States

**Keywords:** prefrontal cortex, adaptive decision making, reward guided choice, action outcome contingency, mediodorsal nucleus, intralaminar nuclei, anterior cingulate cortex, ventral pallidum

## Abstract

Medial prefrontal cortex (mPFC) interacts with distributed networks that give rise to goal-directed behavior through afferent and efferent connections with multiple thalamic nuclei and recurrent basal ganglia-thalamocortical circuits. Recent studies have revealed individual roles for different thalamic nuclei: mediodorsal (MD) regulation of signaling properties in mPFC neurons, intralaminar control of cortico-basal ganglia networks, ventral medial facilitation of integrative motor function, and hippocampal functions supported by ventral midline and anterior nuclei. Large scale mapping studies have identified functionally distinct cortico-basal ganglia-thalamocortical subnetworks that provide a structural basis for understanding information processing and functional heterogeneity within the basal ganglia. Behavioral analyses comparing functional deficits produced by lesions or inactivation of specific thalamic nuclei or subregions of mPFC or the basal ganglia have elucidated the interdependent roles of these areas in adaptive goal-directed behavior. Electrophysiological recordings of mPFC neurons in rats performing delayed non-matching-to position (DNMTP) and other complex decision making tasks have revealed populations of neurons with activity related to actions and outcomes that underlie these behaviors. These include responses related to motor preparation, instrumental actions, movement, anticipation and delivery of action outcomes, memory delay, and spatial context. Comparison of results for mPFC, MD, and ventral pallidum (VP) suggest critical roles for mPFC in prospective processes that precede actions, MD for reinforcing task-relevant responses in mPFC, and VP for providing feedback about action outcomes. Synthesis of electrophysiological and behavioral results indicates that different networks connecting mPFC with thalamus and the basal ganglia are organized to support distinct functions that allow organisms to act efficiently to obtain intended outcomes.

## Introduction

**FIGURE 1:**
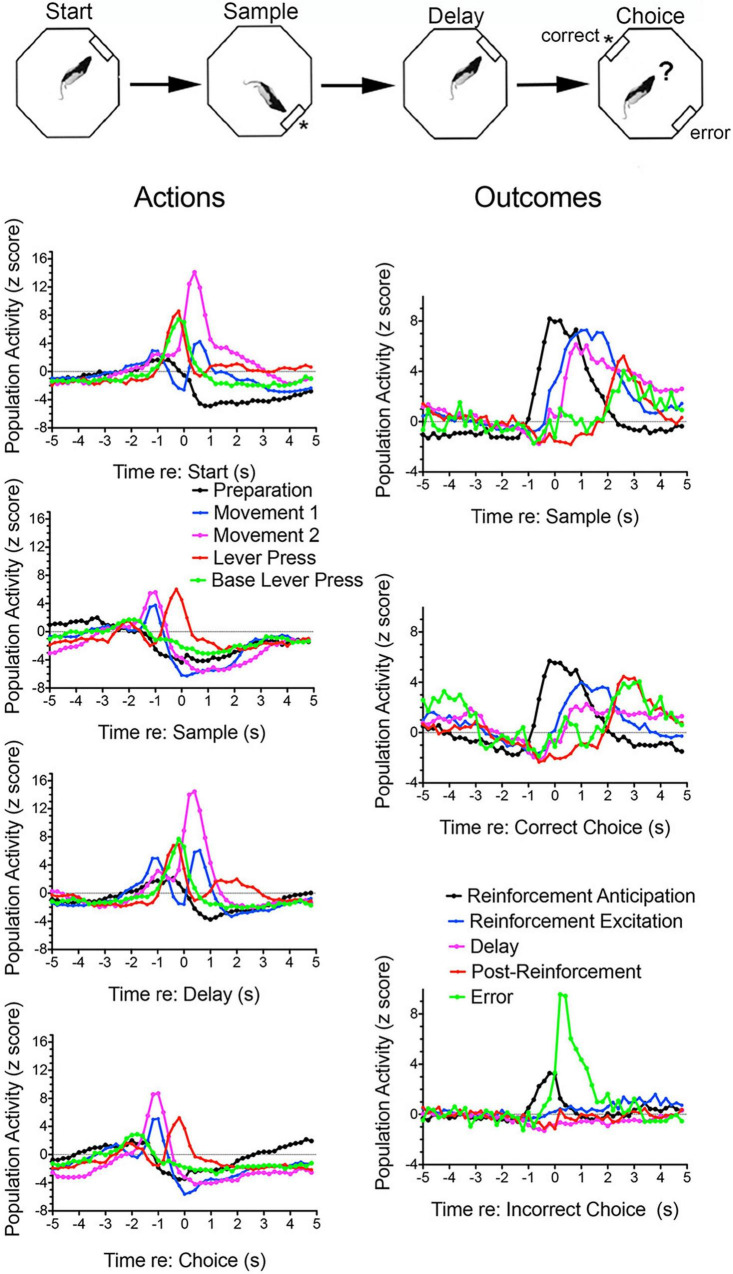
Normalized population histograms for response types observed in mPFC for rats performing the dynamic DNMTP task. The task is illustrated above. Trials begin with a randomly selected lever extending at one of four possible locations (90° apart) for the start response. The start lever retracted when pressed and a lever was then extended 90° to the left or right (randomly selected) of the start lever. This retracted when pressed and water reinforcement delivered (indicated by asterisk) through a spout immediately above the lever. The initial lever was then reinserted for the delay response. This was retracted with the first press after the memory delay ended and levers 90° to the left and right inserted for the choice. These both were retracted when either one was pressed and reinforcement delivered when the lever not extended for the sample was the one pressed (a correct non-matching response). Results are shown for all neuronal responses recorded for each response type with a minimum of 40 trials completed in a 60 m session. These were averaged for individual neurons and normalized so that each response recorded contributed equally to the population function. Error bars represent standard error of the mean. Results are shown for preparation (*n* = 44), movement 1 (before all lever presses, *n* = 97), movement 2 (toward reinforced lever presses, *n* = 32), lever press (*n* = 28), base lever press (start and delay presses only, *n* = 30), reinforcement anticipation (preceding delivery, *n* = 50), reinforcement (following delivery, *n* = 63), delay (*n* = 58), post-reinforcement (when rats disengaged from spouts, *n* = 16), and error (*n* = 4). These population histograms were previously published online (Francoeur and Mair, 2018). ? Represents choice.

The ability to act based on the current incentive value of action outcomes is a defining feature of purposive or goal-directed behavior, one that distinguishes goal-directed responses from stimulus-elicited habits that are unaffected by changing outcome values (Colwill and Rescorla, 1985; Balleine and Dickinson, 1998). Goal-directed actions entail prospective processes to anticipate likely action outcomes, to select and maintain adaptive goals until responses are executed, and to prepare forthcoming motor responses; concurrent processes to guide, coordinate, and monitor ongoing actions and outcomes; and retrospective processes to update information and strategies to guide future responding. These processes are modeled by delayed conditional discriminations, like delayed matching (DMTP) or non-matching (DNMTP) to position, where the rule for selecting a correct (rewarded) choice is indicated by a preceding sample stimulus and executed following a delay. In DMTP and DNMTP the position of the correct choice is indicated by the randomly selected position of the preceding sample response (see Figure 1 for DNMTP example). The central thesis here is that rodent medial prefrontal cortex (mPFC) acts through afferent and efferent connections with multiple thalamic nuclei (Mair et al., 2021) and recurrent networks involving basal ganglia and thalamus (Alexander et al., 1986; Lee et al., 2020; Foster et al., 2021) to control actions motivated by and directed toward an intended outcome. The elaboration of these circuits during vertebrate evolution has been linked to the development of neural systems that underlie more abstract processes of human cognition (Ginsburg and Jablonka, 2010, 2021; Koechlin, 2014; Cisek, 2019).

**FIGURE 2:**
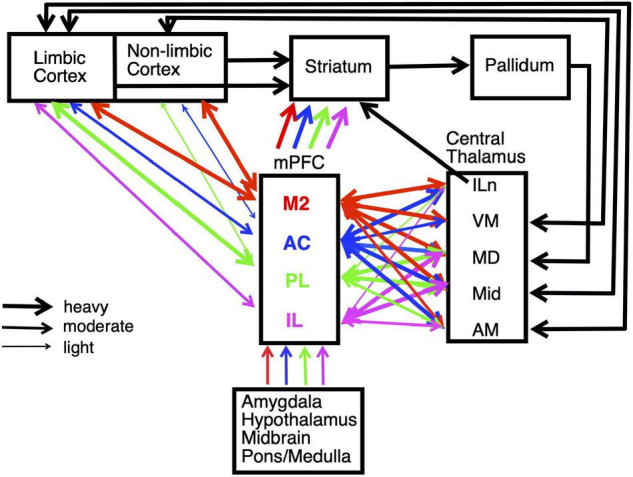
Schematic summarizing the main connections of medial prefrontal cortex (mPFC), including interconnections with striatum and central thalamus. Pathways are color coded to identify afferent and efferent connections of the main subregions of mPFC: secondary motor (M2), anterior cingulate (AC), prelimbic (PL), and infralimbic (IL) cortices. The weight of lines indicates heavy, moderate, or light projections. Unidirectional or bidirectional transmission is indicated by arrowheads. Projections are shown for intralaminar (ILn), ventromedial (VM), mediodorsal (MD), midline (Mid), and anterior medial (AM) nuclei in central thalamus. The division of cortex into limbic and non-limbic regions follows Hoover and Vertes (2007). Estimates of mPFC projection densities rely primarily on Vertes (2002, 2004) and Hoover and Vertes (2007). See text for details.

We focus on behavioral and electrophysiological studies that allow direct comparisons between mPFC, the basal ganglia, and thalamus. Rodent mPFC corresponds to regions of primate cingulate cortex (Vogt et al., 2013; Vogt and Paxinos, 2014). It is at a crossroads between sensory, motor, and limbic systems that give rise to goal-directed behavior (Figure 2). mPFC is commonly divided into five interconnected regions from dorsal to ventral based on anatomical and functional criteria: secondary motor (M2), anterior cingulate (AC), prelimbic (PL), infralimbic (IL), and medial orbital (MO) cortices. All areas of mPFC have afferent and efferent connections with midline, anterior, rostral intralaminar, and mediodorsal (MD) thalamic nuclei as well as limbic or non-limbic cortices, amygdala, hypothalamus, basal forebrain, midbrain, and pons/medulla (Figure 2; Vertes, 2002, 2004; Heidbreder and Groenewegen, 2003; Gabbott et al., 2005; Hoover and Vertes, 2007). M2 receives prominent inputs from sensory, motor, association, and limbic cortices and projects heavily to areas involved with motor control in adjacent motor (M1) cortex, dorsolateral striatum, superior colliculus, oculomotor nuclei, and spinal cord. It has been implicated in motor planning, mapping sensory cues and other antecedent signals to motor actions, and integrating spatial information to guide planned actions (Barthas and Kwan, 2017; Olson et al., 2020; Duan et al., 2021). More ventral areas of mPFC receive progressively less prominent projections from non-limbic cortex and more prominent connections with amygdala and limbic areas of cortex, including hippocampal and parahippocampal areas (Heidbreder and Groenewegen, 2003; Hoover and Vertes, 2007). These have been implicated in multiple functions required for adaptive goal-directed behavior.

## Medial Prefrontal Cortex Supports Prospective, Concurrent, and Retrospective Processes That Give Rise to Adaptive Goal-Directed Behavior

### Anticipating Action Outcomes

Goal-directed responses are guided by the incentive value of anticipated action outcomes (Colwill and Rescorla, 1985; Balleine and Dickinson, 1998). Much of the evidence for this has been obtained by outcome devaluation and instrumental contingency degradation studies. In outcome devaluation animals are trained to make distinct responses for particular outcomes. After initial training one outcome is then devaluated by selective satiation or pairing with lithium chloride to induce illness, and the tendency to make that response is subsequently tested in extinction. If the response is guided by the anticipated value of the outcome, the tendency to respond in extinction should be reduced for devalued outcomes. In instrumental contingency degradation, distinct responses are paired with specific outcomes. After initial training, one outcome is presented non-contingently. If responding is guided by anticipated outcomes, the response associated with the non-contingent outcome should decrease when testing is conducted in extinction. PL lesions made before (but not after) initial training interfere with both outcome devaluation and instrumental contingency degradation (Ostlund and Balleine, 2005; Tran-Tu-Yen et al., 2009; Hart et al., 2018). These findings are consistent with evidence from studies of fear conditioning and drug self-administration that PL is important for encoding the current value of action outcomes thus regulating sensitivity to reinforcement. Earlier studies suggested that IL has effects opposite PL on motivated behavior, with PL promoting goal-directed actions, facilitating reward seeking and fear and IL promoting habit expression, response inhibition, fear suppression, and extinction (see Giustino and Maren, 2015; Moorman et al., 2015; Gourley and Taylor, 2016). More recent results suggest a more nuanced relationship between IL and PL. Shipman et al. (2018) report that both PL and IL influence the expression of goal-directed responses, albeit in distinct ways, with PL affecting the expression of minimally trained and IL the expression of extensively trained actions. Caballero et al. (2019) report these areas both affect reward seeking, but in ways that are inconsistent with the dichotomy of response execution/inhibition.

Bradfield et al. (2015) showed that MO lesions impair outcome-specific Pavlovian-to-instrumental transfer (PIT) and devaluation tests when outcomes are not observable and thus must be retrieved from memory, while sparing outcome-selective reinstatement and contingency degradation conducted in the presence of outcomes. Bradfield et al. (2018) replicated these findings and localized them to anterior regions of MO. Lichtenberg et al. (2021) used chemogenetic methods to show that projections from MO to the basolateral amygdala (BLA) are important for predicting rewards from environmental cues in outcome-specific PIT and for inferring incentive value in devaluation tests while projections from BLA to MO are needed to infer incentive value but not to identify expected rewards.

Although questions remain about their precise roles it seems clear that PL, IL, and MO are important for anticipating the incentive value of potential action outcomes. Neurophysiological recordings of brain activity have confirmed that neurons in these areas fire in anticipation of action outcomes and provided evidence that this information is also represented in other areas that give rise to goal-directed behavior. Human functional magnetic resonance imaging (fMRI) studies show that anticipation of monetary awards is associated with cortical activation in midcingulate/supplementary motor and insular areas distinct from areas of anterior and posterior cingulate cortices activated during award delivery (Jauhar et al., 2021). Electrophysiological studies, primarily in rats and monkeys, have described neuronal activity that signals reward prediction in dorsal and ventral areas of mPFC as well as MO and BLA (Euston et al., 2012; Bissonette and Roesch, 2016). Reward-predictive sensory cues produce short latency responses in PL neurons in trained rodents that are not observed for irrelevant stimuli or for reward-predictive stimuli in naïve animals (Pinto and Dan, 2015; Otis et al., 2017; Le Merre et al., 2018). A number of reports have described neuronal responses that anticipate reinforcement in mPFC of awake, behaving rats and monkeys for several tasks (Pratt and Mizumori, 2001; Alexander and Brown, 2011; Rushworth et al., 2011; Gentry and Roesch, 2018).

**FIGURE 3:**
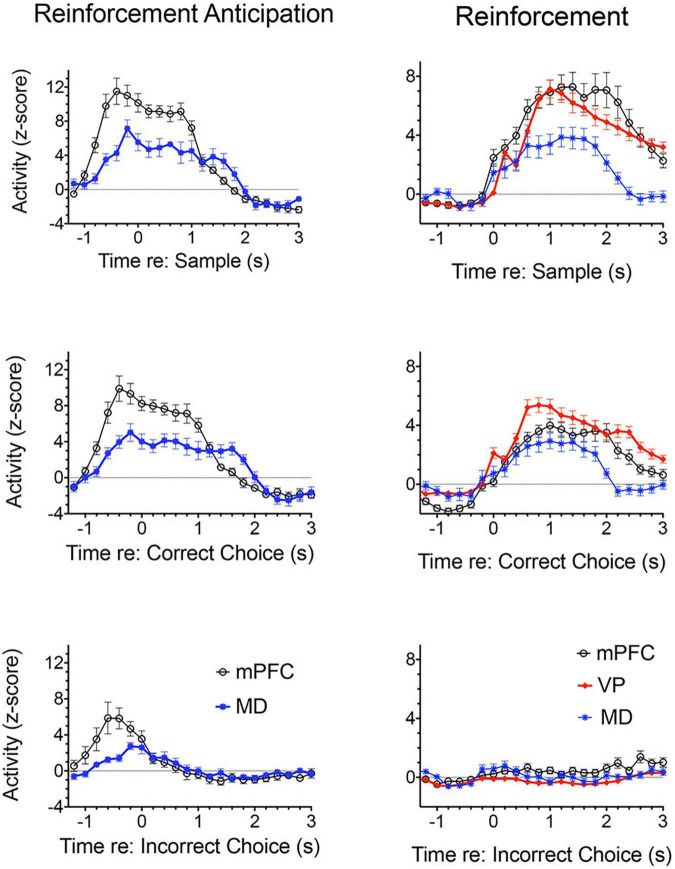
Normalized population histograms responses related to reinforcement delivery in medial prefrontal cortex (mPFC; *n* = 63), the mediodorsal thalamic nucleus (MD, *n* = 71), and ventral pallidum (VP, *n* = 101) and reinforcement anticipation in mPFC (*n* = 50) and MD (*n* = 46). Anticipatory responses were not observed in VP. Anticipatory responses began on average 0.8 s before rewards were normally delivered and ended 0.2 s after reward delivery ended or 0.2 s errors when rewards were not delivered. Reinforcement responses began 0.2 s after reward delivery began and lasted until 1.0 s after reward delivery ended. Results are shown for all neuronal responses recorded for each response type with a minimum of 40 trials completed in a 60 m session. These were averaged for individual neurons and normalized so that each response recorded contributed equally to the population function. Error bars represent standard error of the mean.

Figure 3 shows normalized population histograms based on all mPFC neurons recorded that fired in anticipation of reinforcement (*n* = 50) in rats performing a dynamic DNMTP task (Onos et al., 2016; Francoeur and Mair, 2018). Neuronal firing increased on average 0.8 s prior to times when reinforcement is normally delivered following sample and correct choice responses. These responses persist throughout the subsequent 1.2 s reinforcement event and then drop within 0.2 s after the reward ends. When reward was not delivered following errors anticipatory firing ended abruptly within 0.2 s of the incorrect choice. Although neurons with these anticipatory responses were observed throughout mPFC, they were more concentrated in ventral areas of mPFC including PL regions implicated in behavioral studies of instrumental contingency degradation and selective reward devaluation as important for anticipating the incentive value of action outcomes. A separate population of mPFC neurons was observed that fired in conjunction with reward delivery (Figure 3). These neurons (*n* = 63) exhibited increased activity within 0.2 s after reward delivery began and lasted until 1 s after it ended. They did not respond following unrewarded incorrect choices. It is unclear from timing data whether these responses are related to consummatory activity or reward delivery. Other studies have provided evidence that neurons in orbitofrontal cortex and mPFC encode information about the identity and subjective value of rewards (Wallis and Kennerley, 2010; Cai and Padoa-Schioppa, 2012, 2021; Bissonette and Roesch, 2016; Gentry and Roesch, 2018). Anatomical analyses showed that neurons responding to reward delivery were more uniformly distributed throughout mPFC than those with anticipatory responses (Francoeur and Mair, 2018).

### Prospective Decision Making

**FIGURE 4:**
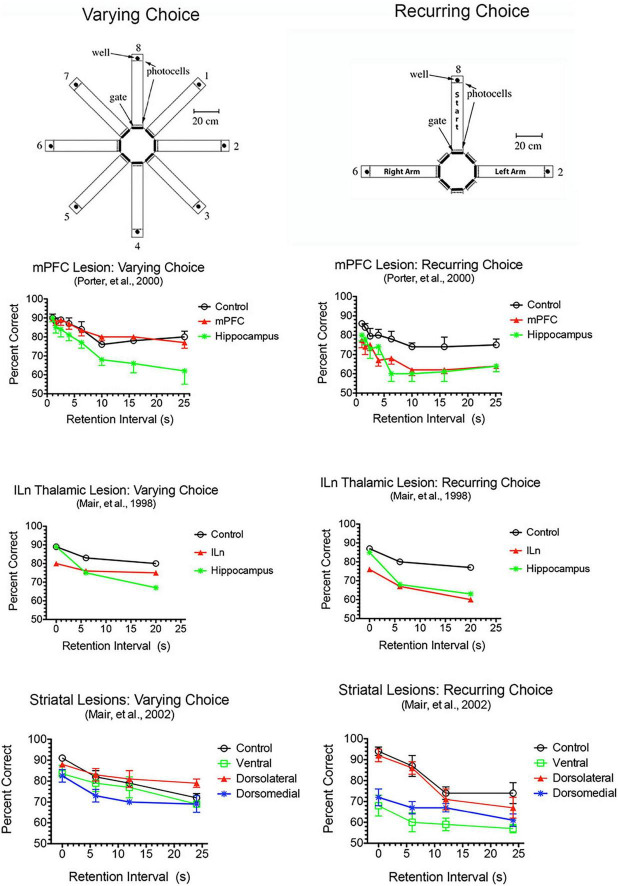
Effects of mPFC, hippocampal, intralaminar thalamic (ILn), and striatal lesions on varying- and recurring-choice DNMTP trained in automated eight arm radial mazes. In varying choice DNMTP arms were selected at random from all available options for start, sample, delay, and choice responses in a lighted room with many visible external cues to favor an allocentric solution and to eliminate the possibility that a correct choice could be determined prospectively. In recurring choice, the same three arms (in a T-configuration) were used on every trial for start/delay and left and right sample/choice responses and mazes were covered and the room darkened to minimize external cues and favor egocentric choice and prospective decision making. mPFC lesions spared varying-choice and produced delay-independent deficits for recurring-choice DNMTP. Hippocampal lesions produced delay-dependent deficits for both versions, consistent with rapid decay of working memory. ILn lesions produced delay-independent deficits for both versions, consistent with the effects of dorsomedial striatal lesions. The ventral striatal lesion group were impaired for recurring-choice DNMTP and were impaired compared to the dorsolateral but not the control group for varying-choice DNMTP. Error bars represent standard error of the mean.

Dorsal mPFC lesions interfere with prospective decision making, where a choice is made and the intention to respond held in memory until intended actions can be carried out (Kesner, 1989; Goto and Grace, 2008; Kesner and Churchwell, 2011; Umeda et al., 2011; Howland et al., 2022). This requires motor response memory to encode, maintain, and retrieve an intention to respond. Dorsal mPFC lesions impair motor response memory in DMTP and DNMTP where choices are defined by egocentric motor responses (R vs. L turns) so that correct responses are defined prospectively before the choice is presented (Porter et al., 2001; Kesner and Churchwell, 2011). This deficit cannot be ascribed to a more general inability to perform conditional discriminations. Complete lesions of mPFC, alone or combined with lateral orbital lesions, impair recurring choice radial maze DNMTP while sparing varying choice DNMTP trained in the same apparatus with carefully matched varying choice procedures (Figure 4). Recurring choice is trained using the same three arms (in a T configuration) on every trial in a darkened room with mazes covered to eliminate external cues and favor an egocentric (response memory) solution. Varying choice uses three arms randomly selected from eight possible arms for each trial with many available external cues: procedures that favor an allocentric solution and do not rely on prospective memory since the location of the correct choice cannot be predicted until the choice phase begins. These deficits also appear to be independent of demands on working memory. They are little affected by the length of the memory delay (Figure 4) and persist with no memory delay in egocentric auditory match-to-position when the discriminative auditory stimulus is present throughout the choice phase (Stevens and Mair, 1998). By contrast, hippocampal lesions produce delay-dependent deficits consistent with working memory impairment for both the varying and recurring choice DNMTP in which deficits increase significantly at longer memory delays (Figure 4).

fMRI analyses show that prospective memory tasks recruit distributed frontoparietal networks in human subjects (Burgess et al., 2011; Cona et al., 2015; Hamm and Mattfeld, 2019). Functional decoding analyses indicate that mPFC is involved in storing delayed intentions to act at a subsequent time (Soon et al., 2008; Momennejad and Haynes, 2013). These results are consistent with well-established evidence that both premotor (Weinrich and Wise, 1982; Pearce and Moran, 2012) and prefrontal (Takeda and Funahashi, 2004; Markowitz et al., 2015) neurons represent prospective information about forthcoming sensory-guided choice responses. Sul et al. (2011) recorded M2 neurons in rats during a maze foraging task and found early responses, 500 ms before rats approach the choice point, that encode information about the forthcoming choice response. Previous studies failed to find evidence for similar action prediction signals in more ventral areas of mPFC for the same task (Sul et al., 2010). These results suggest an important role for M2 encoding response information about impending choices.

To understand the effects of DNMTP decision making on neural activity in mPFC Francoeur and Mair (2020) compared single neuron activity during dynamic DNMTP with a serial lever press task (SLP) in which rats perform the same sequences of actions in the same apparatus with comparable reinforcements (including 30% unreinforced match-to-position “errors”), but with no actual choice between response alternatives. Compared to SLP, DNMTP was associated with enhanced activity of neurons with task-related activity and suppressed activity of neurons with activity unrelated to the task. DNMTP was also associated with preparatory- and delay-related activity not observed with SLP and more frequent responses related to movement and reinforcement. Thus the occurrence of choice in DNMTP appears to engage mPFC producing both adaptive changes in background firing rates and recruitment of neurons with coding properties specifically related to the choice response.

### Preparing to Respond

Multiple brain areas involved in planning and executing actions are active well before movement begins. In human subjects simple self-initiated movements are preceded by a slow rising negative motor-related cortical potential that originates in supplementary and cingulate motor areas 1–3 s before movement. This is followed by a later negative slope phase 400–500 ms before movement onset that originates in premotor and motor areas and then a motor potential coincident with movement initiation that originates in motor cortex (Di Russo et al., 2017).

Motor preparation and learning can be studied with action sequence learning tasks, where subjects perform a repeated sequence of actions. Response time (RT) analyses have revealed two levels of organization in learned action sequences: actions initiating learned sequences or chunks of learned sequences have elevated RTs reflecting the cost of planning an organized sequence ahead of time while actions later in sequences (or chunks) exhibit reduced RTs reflecting the benefits of performing a practiced sequence (Rosenbaum et al., 1984; Kennerley et al., 2004; Bailey and Mair, 2006). Bailey and Mair (2007) examined effects of frontal cortical lesions in rats trained to perform repeated sequences of five nose pokes guided by luminance cues in an array of response ports. Sequence learning was assessed by interference effects when training shifted from learned (repeated) to random sequences of port entries. Lesions damaging M2 cortex alone or in combination with adjacent areas of M1 or AC cortex increased RT for initial nose pokes, indicating an increased planning cost, while sparing the benefits of sequence learning reflected in decreased RT to complete later pokes in learned sequences. Lesions of M1 cortex had no effect on performance. None of the lesions had any effect on initial RT when planning costs were minimized by comparing single nose poke responses that were in repeated vs. randomized locations. Quantitative histological analyses confirmed that the increased planning cost for repeated sequences correlated significantly with damage to M2, but not to M1 or AC cortices.

While fMRI analyses have revealed preparatory activity in multiple brain regions, including motor, somatosensory, and parietal regions, only signals originating in contralateral supplementary motor and premotor regions consistently predict the type of sequential finger movement executed (Nambu et al., 2015). AC cortex has also been implicated in motor intention when finger presses are cued by motor stimuli (Kuhns et al., 2017). Single unit recordings in human subjects reveal progressive recruitment of neurons with increasing activity preceding self-initiated movements in supplementary motor area and AC beginning 1,500 ms before subjects report making a decision to move (Fried et al., 2011). Single neuron recordings have found similar preparatory increases in activity preceding self-initiated movements in monkey motor and premotor cortex and shown that these responses encode specific movement-related information (Riehle and Requin, 1989; Churchland et al., 2010). Similar results have been observed in recordings of large populations of neurons in premotor cortex in mice in conjunction with perturbations of activity that demonstrate a link to behavior (Li et al., 2015; Svoboda and Li, 2018). Totah et al. (2009, 2013) have described synchronous preparatory activity in rodent AC and PL related to stimulus presentation and motor responses preceding stimulus-guided actions in the 5-choice task. Similarly we have observed preparatory activity in mPFC prior to start responses initiated by rats in the dynamic DNMTP task (Onos et al., 2016; Francoeur and Mair, 2018). These responses consist of activity ramping up beginning on average from 2.4 s and ending 0.6 s prior to start lever presses at the beginning of trials. A smaller response is also observed from 1.3 to 0.7 s prior to delay lever presses, consistent with rats chunking DNMTP trials into sample and choice phases. The time course of these responses is consistent with motor-related cortical potentials and single unit activity in human subjects observed before self-initiated movements (Fried et al., 2011; Di Russo et al., 2017). Anatomical analyses revealed a significant trend for preparatory responses to be distributed in dorsal areas of mPFC including M2 and AC cortices (Francoeur and Mair, 2018).

### Sensory-Guided Responding

**FIGURE 5:**
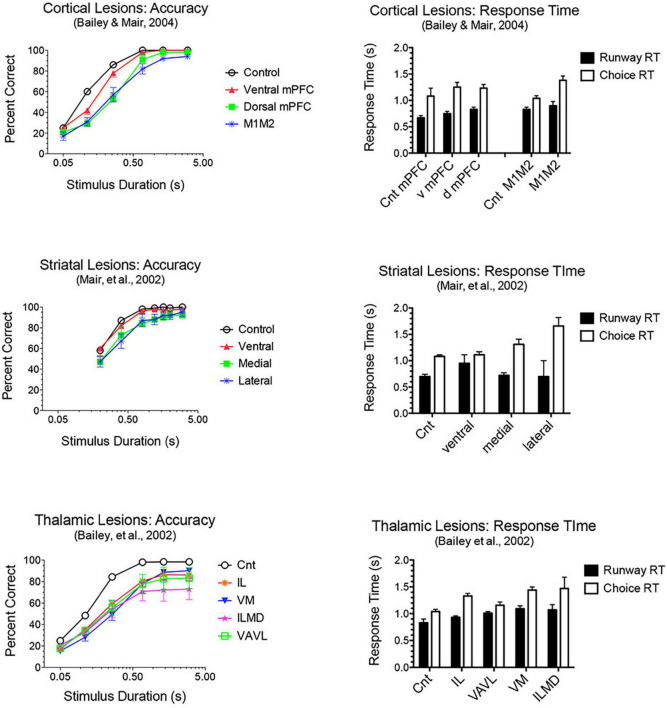
Effects of frontal cortical, striatal and thalamic lesions on visuospatial reaction time (VSRT). Rats performed an observational (runway) response, traveling down a runway after pressing a lever to start the trial, so that they entered the test chamber facing an array of response ports. Entering the test chamber triggered a brief luminance in one of the ports in which water reward was delivered if rats made a nose poke in that port first within a 3.0 s limited hold. Choice accuracy is plotted as a function of stimulus duration (varied randomly from trial to trial). Response time (RT) is plotted for the observational runway response, performed without modification at the start of each trial, and for the stimulus guided choice response. A similar pattern of impairment was observed for lesions of M1M2 motor cortex, dorsolateral striatum, and intralaminar thalamic nuclei (IL): stimulus duration dependent impairment of choice accuracy coupled with an increase in choice, but not runway, RT.

Adaptive responding requires constant updating of sensory information to guide ongoing actions in dynamic environments. M2 plays a critical role in flexibly mapping sensory signals to motor actions, consistent with its prominent connections to sensory and association cortices and motor control areas (Barthas and Kwan, 2017; Olson et al., 2020; Duan et al., 2021). Earlier studies showed that lesions of M2 cortex in rats (Passingham et al., 1988) or premotor cortex in monkeys (Petrides, 1982; Halsband and Passingham, 1985) impair conditional discriminations where a visual cue indicates which of two actions will be reinforced. The effects of mPFC lesions extend to simple sensory-guided responses in visuospatial reaction time (VSRT) tasks (Figure 5). Here rats perform an observational response (pressing a lever and traveling down a runway) and then enter a test chamber facing an array of choice response ports. Test chamber entry triggers a luminance cue (0.2–3.0 s in duration) in the S+ port, indicating the location where reward is delivered following a nose poke within 3.0 s of triggering the cue (the limited hold). Lesions damaging dorsal or ventral mPFC or adjacent areas of M1M2 motor cortices decreased accuracy for brief cues (≤0.8 s) and increased RT for choice responses (Burk and Mair, 2001b; Bailey and Mair, 2004, 2007). The increase in choice RT cannot be explained by decreased accuracy. Choice RT was increased at long stimulus durations where performances had few errors and was not exacerbated when accuracy was diminished by task manipulations. M1M2 lesions did not affect runway RT where habitual responses involving comparable movement distances are repeated without modification on each trial. These results are consistent with other evidence that dorsal mPFC is important for sensory-guided motor function and more ventral mPFC for sensory attention (Brown et al., 1991; Muir et al., 1996; Bussey et al., 1997; Birrell and Brown, 2000). The effects of M1M2 lesions on VSRT are consistent with deficits observed with lesions of dorsolateral striatal areas innervated by these areas of cortex: impaired choice accuracy and RT without significant effect on runway RT (Figure 5).

### Coordinating and Monitoring Ongoing Actions and Outcomes

Convergent evidence indicates that cingulate areas of mPFC monitor current actions and outcomes, provide error feedback and information about reward value and manage conflict when there is competition between potential actions or strategies (Ridderinkhof et al., 2004; Holroyd and Coles, 2008; Kennerley et al., 2011; Heilbronner and Hayden, 2016; Li et al., 2019; McLaughlin et al., 2021). Contingency degradation and reward devaluation studies have confirmed the importance of mPFC in monitoring information about current incentive value and action-outcome contingencies (see above). Populations of mPFC neurons exhibit activity modulated by ongoing actions and outcomes consistent with a role in monitoring this information during goal-directed behavior (Jung et al., 1998; Chang et al., 2002; Euston and McNaughton, 2006; Totah et al., 2009, 2013; Hayden and Platt, 2010; Alexander and Brown, 2011; Rushworth et al., 2011; Horst and Laubach, 2012). Figure 1 shows normalized population histograms observed for mPFC neurons during dynamic DNMTP trials. These include results from all neurons firing during all periods of movement, movements preceding rewards, lever press responses, reward delivery, delay periods following rewards, errors when expected rewards are not delivered, and disengagement from reinforcement. Importantly, some of these responses occur in restricted spatial locations and thus represent contextual information about locations where the behavioral events occur (Onos et al., 2016). This was demonstrated by concurrent spatial mapping of activity and temporally defined event-related analyses using a dynamic DNMTP task where trials began at randomly selected locations so that behavioral events were disambiguated from spatial location from trial to trial.

Lesions or inactivation of mPFC affect the ability of organisms to adapt when contingencies between stimuli, actions, and outcomes are changed during extinction learning (Quirk and Mueller, 2008; Peters et al., 2009; Caballero et al., 2019; Porter and Sepulveda-Orengo, 2020; Green and Bouton, 2021; Russo et al., 2021). Electrophysiological recordings have identified transient post-decision signals in cingulate cortices of humans, monkeys, and rodents that indicate when rewards are received, errors are made, or expected reward not delivered (Gemba et al., 1986; Ito et al., 2003; Botvinick et al., 2004; Matsumoto et al., 2007; Totah et al., 2009; Horst and Laubach, 2012; Li et al., 2019; Kao et al., 2020). Figure 1 shows error-related responses recorded after unreinforced incorrect choice responses in the DNMTP task. Francoeur and Mair (2020) observed an analogous response during the SLP task. Here error-like responses were observed for the 30% of trials when reward was not delivered for the fourth press (to simulate DNMTP errors). This suggests that error responses signal the lack of expected reward, not the selection of an incorrect choice (since there was no choice in SLP). DNMTP errors were also signaled by reinforcement anticipation responses which ended abruptly (within 0.2 s) of unreinforced errors, compared to 1.4 s following reinforced correct responses (Figure 3).

### Updating Memory

To respond adaptively in dynamic contexts organisms must constantly update information about environmental conditions and action-outcome contingencies across short and long timescales. mPFC is interconnected with important memory systems and has functional properties that seem well suited for this purpose (see Euston et al., 2012; Monosov et al., 2020). mPFC is necessary for both extinction learning when contingencies change (Quirk and Mueller, 2008; Peters et al., 2009; Caballero et al., 2019; Green and Bouton, 2021; Russo et al., 2021) and for acquisition of new action-outcome associations in goal-directed behavior (Ostlund and Balleine, 2005; Tran-Tu-Yen et al., 2009; Hart et al., 2018). mPFC has also been linked to working memory, promoting adaptive responding by allowing organisms to hold and manipulate response-related information over brief periods of time while monitoring and navigating dynamic environments. Early treatments emphasized the role of prefrontal cortex in working memory and identified prolonged neuronal firing as a mechanism to represent information over short memory delays (Fuster and Alexander, 1971; Goldman-Rakic, 1995). More recently questions have been raised about what role this persistent firing plays in working memory, the extent to which this represents retrospective sensory vs. prospective motor information, and the extent to which working memory is a function shared with other areas of neocortex (Funahashi, 2013; Constantinidis et al., 2018; Lundqvist et al., 2018; Miller et al., 2018). Lesion studies (see above) have shown effects of prefrontal lesions on some, but not all delayed conditional discriminations used to measure working memory, consistent with the view that working memory is not solely the province of prefrontal cortex (Koger and Mair, 1994; Porter et al., 2001; Kesner and Churchwell, 2011; Scott and Mishkin, 2016; Roussy et al., 2021). Figure 1 shows normalized population histograms for delay-related responses in mPFC of rats performing a DNMTP task. These begin during reinforcement delivery and are more substantial following reinforced sample responses than correct choice responses. The sample-related response corresponds to the critical period when information needs to be remembered until a choice is selected, although it is not clear what specific information is represented by this activity. Similar responses have been found for rodent mPFC neurons firing persistently during memory delays without a clear correlation with task-relevant discriminative information (Jung et al., 1998; Baeg et al., 2003; Euston and McNaughton, 2006; Euston et al., 2012; Horst and Laubach, 2012; Liu et al., 2014).

Longer-term memories promote adaptive responding by gradually integrating information across time, a function that allows organisms to adjust to lasting changes in the external environment or response contingencies. All areas of mPFC receive projections from the hippocampal-entorhinal cortex network and the BLA (Figure 2; Hoover and Vertes, 2007), important for gradual long-term memory consolidation. Systems memory consolidation theory suggests that labile memories are transformed into a more permanent and stable form required for long-term memory by changes in brain circuitry involving these three areas (Kitamura et al., 2017; Tonegawa et al., 2018; Takehara-Nishiuchi et al., 2020). Bontempi et al. (1999) first reported that metabolic activity was higher in mPFC and other cortical areas following remote (25 days) than recent (5 days) spatial memory testing, while activity in the hippocampal formation showed the opposite trend. Subsequent studies confirmed that reversible inactivation of mPFC affects retrieval of remote, but not recent, memories for contextual fear conditioning (Frankland et al., 2004), spatial memory (Teixeira et al., 2006), trace eyeblink conditioning (Takehara-Nishiuchi et al., 2006) and paired associate memory (Wang et al., 2012). Although these retrieval deficits occur after a period of weeks, mPFC undergoes rapid changes in neuronal activity, gene expression, and synaptic structure beginning at the time of initial training and lasting over a period of at least 2 weeks (Tonegawa et al., 2018; Takehara-Nishiuchi et al., 2020). These results suggest that systems consolidation is a lengthy, continuous process. Recent evidence has also shown that mPFC is unique among cortical areas activated during episodic memory formation and retrieval, as a location where inhibition of hippocampal-entorhinal inputs during learning blocks subsequent longer-term learning-related changes in neuronal function (Kitamura et al., 2017; Tonegawa et al., 2018). These results suggest that mPFC plays a critical role in formation and retrieval of remote memories while the hippocampal formation is more important for forming rapid associations between ongoing events and stabilizing long-term memory traces in mPFC and other areas of neocortex (Tonegawa et al., 2018; Takehara-Nishiuchi et al., 2020).

## Central Thalamic Nuclei Interact With Medial Prefrontal Cortex and the Basal Ganglia in Large Scale Networks That Underlie Adaptive Goal-Directed Behavior

All areas of mPFC have afferent and efferent connections with multiple nuclei in central thalamus (Figure 2). These are higher-order thalamic nuclei that receive their main driver inputs from cortex and appear organized to serve as elements in large-scale networks that support specific aspects of adaptive goal-directed behavior (see above; Mitchell et al., 2014; Sherman, 2016). MD is strongly excited by driver (layer 5) and more prominent modulatory (layer 6) projections from mPFC. It is the main source of focal thalamic projections to middle layers of mPFC and sparser diffuse projections to layer I. These thalamocortical projections activate excitatory networks and feedforward inhibition in mPFC (Groenewegen, 1988; Kuroda et al., 1996, 1998; Xiao et al., 2009; Collins et al., 2018). Recent evidence indicates that MD enhances cortical connectivity and regulates signal processing properties of mPFC neurons through genetically defined subpopulations of thalamocortical neurons that compensate for uncertainty related to low signals or high levels of noise (Schmitt et al., 2017; Mukherjee et al., 2021).

The central lateral (CL), paracentral (PC), and central medial (CM) rostral intralaminar nuclei project to distinct areas of mPFC and striatum that are connected by corticostriatal projections. These appear organized to control cortical-basal ganglia networks and thus the selection of goals, actions, and sensory signals (Groenewegen and Berendse, 1994; Grillner et al., 2005; Mannella et al., 2016; Kato et al., 2018). The ventral medial (VM) nucleus has reciprocal connections with AC, M2, and adjacent sensorimotor cortex consistent with initiation and control of integrative motor responses (Vertes, 2002; Hoover and Vertes, 2007; Collins et al., 2018; Takahashi et al., 2021). The paraventricular (PV), paratenial (PT), reuniens (Re) and rhomboid (Rh) midline nuclei and the interoanteromedial (IAM) and anterior medial (AM) nuclei have afferent and efferent connections with mPFC and with other cortical and subcortical limbic structures. These have been implicated in limbic functions including affective behaviors, spatial and non-spatial learning, recent and remote memory, behavioral flexibility, and spatial navigation (Aggleton and Nelson, 2015; Vertes et al., 2015; O’Mara and Aggleton, 2019).

**FIGURE 6:**
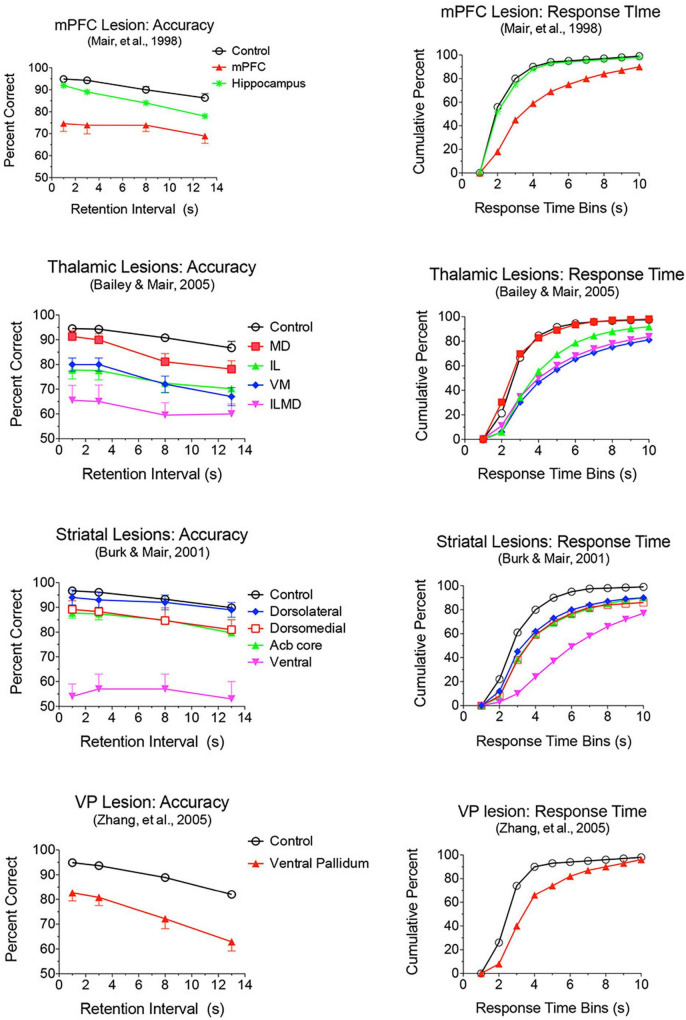
Effects of mPFC, hippocampus, thalamic, striatal, and ventral pallidal lesions on DMTP trained in operant chambers with 3 retractable levers: two on the front wall for sample and choice responses and one on the back wall to initiate trials and to force rats to disengage from front wall levers during memory delays. Accuracy is plotted as percent correct as a function of memory delay and response time (RT) as cumulative functions showing the percentage of choice responses made in 1 s bins after the end of the memory delay. Hippocampal and mediodorsal thalamic (MD) lesions produced delay-dependent impairment of accuracy and had no effect on RT. Dorsolateral lesions had no significant effect on accuracy or RT. Dorsomedial and nucleus accumbens (Acb) core produced delay independent deficits in accuracy, significantly and had no significant effect on RT. mPFC, ventral pallidum, ventral striatum, and Intralaminar (IL), ventromedial (VM), and combined intralaminar/mediodorsal (ILMD) thalamic lesions produced delay-independent deficits in accuracy and increased median RT significantly.

Lesion and inactivation studies have confirmed the dependence of mPFC function on distinct contributions of different thalamic nuclei (see Mair et al., 2021 for review). MD supports learning and decision making, particularly tasks that require rapid trial-by-trial learning and complex decision making (see Mitchell and Chakraborty, 2013; Mitchell, 2015). MD lesions produce deficits similar to mPFC lesions for the acquisition of goal-directed actions but have limited effects on other measures of mPFC function. Pre-training lesions of MD and PL cortex have common effects on the acquisition of action-outcome associations tested by outcome devaluation and contingency degradation (Corbit et al., 2003; Ostlund and Balleine, 2008). Alcaraz et al. (2018) used chemogenetic methods to show that projections from mPFC to MD are necessary for adapting to current incentive value in outcome devaluation but not action-outcome contingencies while MD to mPFC projections are needed for both. MD lesions have limited effects on egocentric DNMTP and DMTP, producing delay-dependent decreases in response accuracy without affecting response speed (Figure 6; Bailey and Mair, 2005; Bolkan et al., 2017). By contrast mPFC lesions produce more substantial, delay-independent deficits that affect both response speed and accuracy for DMTP and DNMTP (Young et al., 1996; Mair et al., 1998; Porter et al., 2000). Like mPFC lesions, MD lesions spare allocentric radial maze DNMTP where the correct response is not revealed until the choice phase begins (Bailey and Mair, 2005). Bolkan et al. (2017) used optogenetic methods to demonstrate delay-dependent effects of MD to mPFC projections on working memory in a T-maze DNMTP task, which they related to suppression of delay-related neuronal activity in mPFC by this treatment. Interestingly, while inhibition of MD to mPFC projections disrupted performance when applied during the delay, but not during the sample or choice phases. By contrast inhibition of mPFC to MD projections impaired performance during the choice phase only. While these results support a role for MD to mPFC projections in working memory it is also possible that they reflect the hypothesized role of MD regulating signal processing properties of mPFC neurons by enhancing uncertain signals at longer retention intervals (Mukherjee et al., 2021).

Lesions damaging the rostral intralaminar nuclei (CL, PC, and CM) have effects comparable to mPFC lesions on DMTP and DNMTP: producing delay-independent deficits for egocentric DMTP and DNMTP that affect both speed and accuracy of choice responses with more limited effects on allocentric radial maze DNMTP (Figure 6; Knoth and Mair, 1991; Young et al., 1996; Burk and Mair, 1998; Mair et al., 1998; Bailey and Mair, 2005; Mitchell and Dalrymple-Alford, 2006). Intralaminar lesions also resemble mPFC lesions in their effects on sensory-guided responding in the VSRT task: decreased accuracy for brief cues, increased RT for choice responses at all stimulus durations, and relative sparing for RT of observational responses (Figure 5; Burk and Mair, 2001b). Lesion studies have revealed parallel effects of striatal lesions on DMTP, DNMTP, and VSRT tasks (Figures 4–6; see below) in keeping with anatomical evidence that the intralaminar nuclei are organized to regulate interactions between mPFC and striatum (Groenewegen and Berendse, 1994). Lesions of the VM nucleus also produce delay-independent impairment of DMTP choice accuracy and speed and VSRT accuracy (for short stimulus durations) and speed (Figures 5, 6). Unlike intralaminar lesions, VM lesions had substantial effects on VSRT RT for both observational and choice responses that were significantly greater than for intralaminar lesions. These broad and sizeable effects on RT are consistent with evidence that VM is a critical node in motor preparation. It is strongly driven by corticothalamic projections from mPFC (Collins et al., 2018) and transmits an urgency or vigor signal from basal ganglia to layer I of M2, AC, and adjacent sensorimotor cortex that facilitates the initiation of cue-triggered motor responses (Bosch-Bouju et al., 2013; Guo et al., 2017; Takahashi et al., 2021).

The anterior thalamic AM and IAM nuclei are important nodes in pathways linking the hippocampal system with PL and AC areas of mPFC (Aggleton and Nelson, 2015). AM and IAM lesions affect measures of allocentric memory spared by mPFC and MD lesions (Warburton et al., 1997; Porter et al., 2000; Mair et al., 2003; Bailey and Mair, 2005; Wolff et al., 2008), deficits that have been dissociated with the effects of lesions damaging MD and intralaminar nuclei on egocentric DMTP (Mitchell and Dalrymple-Alford, 2006) and a spatial outcome devaluation task (Alcaraz et al., 2016). This suggests that AM and IAM support spatial memory functions that are presumably influenced by mPFC but not disrupted by mPFC lesions. Re and Rh are reciprocally connected to mPFC and send projections to hippocampus and subiculum hypothesized to coordinate the synchrony of these areas during spatial memory tasks (Vertes et al., 2015; Hallock et al., 2016). Behavioral analyses have shown that lesions or inactivation of these nuclei affect spatial memory tasks that depend on the integrity of both mPFC and the hippocampal system (Hembrook and Mair, 2011; Hembrook et al., 2012; Cholvin et al., 2013), disrupting both encoding and retrieval of spatial and contextual memories (Ramanathan et al., 2018; Rahman et al., 2021; Schwabe et al., 2021). Other studies have provided evidence that Re and Rh are important for long-term and remote memory consolidation and retrieval (Loureiro et al., 2012; Barker and Warburton, 2018; Ferraris et al., 2021), processes that also depend on interactions of mPFC with the hippocampal formation (see above). The dorsal midline nuclei, PV and PT, have prominent connections with PL, IL, and MO cortices as well as agranular insular and entorhinal cortices, subiculum, dorsal and ventral striatum, and the extended amygdala (Vertes and Hoover, 2008). Lesion and inactivation studies indicate that these nuclei support reward seeking behaviors and emotional processing but not the spatial memory processes affected by Re and Rh (Barson et al., 2020; McGinty and Otis, 2020; Mair et al., 2021).

**FIGURE 7:**
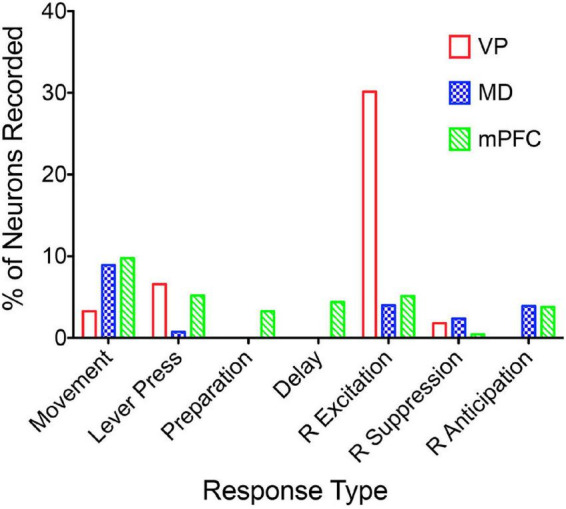
The percentage of units recorded that meet the criteria for an isolated neuron that also meet criteria for specific response types (see Francoeur and Mair, 2018). Movement and lever press types are combined into single categories. Results are compared for ventral pallidum (VP), mediodorsal thalamus (MD), and medial prefrontal cortex (mPFC).

Miller et al. (2017) examined responses of MD neurons during the same dynamic DNMTP task used earlier to characterize response properties of mPFC neurons (Onos et al., 2016; Francoeur and Mair, 2018). They found populations of neurons with many of the same responses related to actions and outcomes observed in mPFC (Figure 7), including subpopulations that represent contextual information by responding to specific events in restricted spatial locations. These similarities are not surprising given the strong reciprocal connections between mPFC and MD. There were several important differences. MD lacks neurons with responses related to preparation, memory delay, and lever press responses and has a significantly larger number in which activity is suppressed during reinforcement. The large number of reinforcement suppression responses is consistent with the robust GABAergic projection from VP signaling reinforcement (see below). The lack of MD neurons firing during preparation, prospective decision making delays, and lever press responses suggest that there may be some selectivity in the information transferred by corticothalamic projections from mPFC to MD. VM may be a more likely target for mPFC neurons exhibiting preparatory responses. As noted above VM is strongly driven by mPFC and facilitates the initiation of cue-triggered motor responses in sensorimotor cortex (Bosch-Bouju et al., 2013; Guo et al., 2017; Collins et al., 2018; Takahashi et al., 2021). Although similar numbers of MD and mPFC neurons exhibit reinforcement anticipation responses that signal impending reward, these responses occur earlier and are more robust in mPFC than in MD, trends not seen for other reward-related responses (Figure 3; Miller et al., 2017). Taken together these results are consistent with evidence (reviewed above) that prospective signals related to predicting action outcomes, prospective decision making, and motor preparation originate in cortical networks involving mPFC.

Francoeur et al. (2019) examined the influence of central thalamus on mPFC by inactivating central thalamus while recording mPFC neurons in rats performing the dynamic DNMTP task used earlier to characterize response properties of mPFC and MD neurons (Onos et al., 2016; Miller et al., 2017; Francoeur and Mair, 2018). Thalamus was inactivated by microinjecting muscimol at sites and doses shown to impair speed and accuracy of responding for DMTP (Mair and Hembrook, 2008) and VSRT (Newman and Mair, 2007) when administered bilaterally. Single cells were recorded across three consecutive days so that activity could be compared for day 2 inactivation with days 1 and 3 when thalamus was not inactivated. Inhibition and recording were done unilaterally to avoid disrupting behavioral performance during recording studies. Thalamic inactivation decreased spontaneous activity for some mPFC neurons and increased it for others, consistent with the well-established excitatory and inhibitory effects of central thalamic inhibition on mPFC activity (Cruikshank et al., 2010, 2012; Delevich et al., 2015; McCormick et al., 2015; Hu and Agmon, 2016). Quantitative analyses revealed a broad suppression of event-related responses in mPFC that did not interact with response type analyzed, effect on spontaneous firing (increased, decreased, or unchanged), dorsal vs. ventral location in mPFC, or muscimol dose tested. These results provide direct evidence that behavioral impairments observed for DMTP (Mair and Hembrook, 2008) and VSRT (Newman and Mair, 2007) with comparable inactivation treatments (applied bilaterally) are associated with a general suppression of mPFC responses related to actions and outcomes.

## The Basal Ganglia Integrate Cortical, Limbic, and Thalamic Inputs Through Multiple Subnetworks

Striatum, the input side of the basal ganglia, receives projections from virtually all areas of cerebral cortex and the limbic system (Figure 2; McGeorge and Faull, 1989; Sesack et al., 1989; Groenewegen et al., 1990). These converge with projections from intralaminar thalamic nuclei on dendrites of medium spiny neurons, which make up about 90% of striatal neurons and provide striatopallidal and striatonigral projections that transmit information about motor commands, sensory data, and incentive value of action outcomes (Groenewegen and Berendse, 1994; Smith et al., 2004, 2011, 2014; Doig et al., 2010; Graybiel and Grafton, 2015; Johansson and Silberberg, 2020; Foster et al., 2021). In rodents AC, PL, IL, and MO areas of mPFC have dense projections to specific areas of dorsomedial and ventral striatum that overlap with each other and with dense projections originating in other areas of prefrontal cortex. M2 stands out as having relatively segregated dense projections to dorsolateral striatum with only a 5% area of overlap with dense projections from AC. Interactions between corticostriatal projections from M2, AC, PL, IL, and MO are expanded by diffuse terminal fields that surround areas receiving dense projections from each of these areas (Mailly et al., 2013; Haber, 2016; Heilbronner et al., 2016; Hintiryan et al., 2016). Large-scale mapping has revealed numerous functional domains in rodent striatum based on convergence and divergence of corticostriatal and thalamostriatal projections (Hintiryan et al., 2016; Hunnicutt et al., 2016). These are organized as subnetworks that are preserved through pallido/nigral and thalamic nodes and back as parallel closed loop circuits to the mPFC corticostriatal neurons from which they originate (Alexander et al., 1986; Lee et al., 2020; Foster et al., 2021). The evidence of multiple cortico-basal ganglia-thalamic subnetworks suggests that there is a high degree of functional heterogeneity within striatum.

Lesion studies have demonstrated functional specialization at the level of the broad channels of information flow in dorsolateral, dorsomedial, and ventral striatum. Mair et al. (2002) found a double dissociation in striatum where dorsolateral striatal lesions increase RT to respond to the trial-specific location of visual stimuli in VSRT while sparing radial maze DNMTP. By contrast, ventral and dorsomedial striatal lesions spare VSRT and impair radial maze DNMTP (Figures 4, 5). Importantly dorsolateral lesions do not significantly affect RT for observational responses which are performed in a stereotyped fashion at the start of each VSRT trial. These results are consistent with evidence that ventral striatal and ventral pallidal lesions increase RT and decrease accuracy for an operant (lever press) DMTP task that is spared by dorsolateral striatal lesions (Figure 6; Burk and Mair, 2001a; Zhang et al., 2005) and that dorsolateral lesions increase RT for visually guided responding in other tasks (Brasted et al., 1999; Rogers et al., 2001). These deficits are of note because of evidence linking them to corticostriatal projections from mPFC and thalamostriatal projections from the intralaminar nuclei. Thus, lesions of mPFC or of the intralaminar thalamic nuclei produce similar delay-independent deficits for recurring choice DNMTP and operant DMTP, increase RT for DMTP, and increase choice RT for the VSRT task (Figures 4–6; Burk and Mair, 1998, 2001b; Porter et al., 2000). Mediodorsal thalamic lesions adjacent to the intralaminar nuclei have distinct effects on DMTP, producing smaller delay-dependent deficits for accuracy with no effect on RT (Figure 6; Burk and Mair, 1998; Bailey and Mair, 2005). Comparison of more discrete mPFC lesions reveal topographically specific effects: lesions damaging PL or AC produce deficits like ventral or dorsomedial striatal lesions affecting recurring choice radial maze DNMTP and sparing VSRT choice RT. By contrast lesions of M1M2 cortex produce deficits like dorsolateral striatal lesions increasing VSRT choice RT and sparing recurring choice DNMTP (Figures 4, 5; Bailey and Mair, 2004).

Dorsolateral and dorsomedial striatum have also been dissociated for tasks used to distinguish goal-directed and habitual action control. Treatments disrupting dorsolateral striatum increase sensitivity to outcome devaluation and contingency degradation, interfere with execution of species-specific and learned sequential actions, and bias animals toward spatial rather than response-related cues in navigation while disruption of dorsomedial striatum blocks sensitivity to outcome devaluation and contingency degradation, decreases dependence on spatial navigation cues and increases reliance on habit learning (Cromwell and Berridge, 1996; Graybiel and Grafton, 2015; Amaya and Smith, 2018; Turner et al., 2022). These findings are often described as functional specializations of dorsomedial striatum for goal-directed behavior in early stages of instrumental conditioning that shifts to dorsolateral control if conditions lead to more automatic habitual responding. Seen through the lens of recent large-scale mapping studies, it may be more parsimonious to reframe this hypothesis in terms of multiple parallel pathways connecting the basal ganglia with thalamus and cortex. Thus dorsolateral striatum, with prominent inputs from motor and sensory cortices and return closed loop projections to M1 and M2 (Hintiryan et al., 2016; Aoki et al., 2019; Lee et al., 2020; Foster et al., 2021) seems organized to support M2 in guiding movements and actions based on information about environmental conditions, including antecedent sensory cues (Barthas and Kwan, 2017; Olson et al., 2020). While linking actions to antecedent cues is integral to habitual S-R learning, it is also important for non-habitual functions related to motor planning, sensory-guided choice, and navigation.

The multiple striatal subnetworks revealed by large-scale mapping studies indicate that striatal specialization extends beyond the broad division into dorsomedial, dorsolateral, and ventral domains. Hintiryan et al. (2016) identified 29 distinct functional domains in mouse dorsal striatum that represent extensive integration and interaction of inputs from previously identified intracortical networks. Although patterns of cortical afference provide important clues about functions mediated by different striatal subnetworks these remain to be fully elaborated. The auditory domain in posterior dorsal striatum provides a good example of a functional subnetwork. This area receives projections from auditory neurons in cortex and thalamus that have distinct effects on responses of striatal neurons to sound and have both been shown to be critical for auditory discrimination learning and associated changes in plasticity of synapses on medium spiny neurons (Znamenskiy and Zador, 2013; Xiong et al., 2015; Chen et al., 2019). In contrast to other areas of dorsal striatum, optogenetic stimulation of auditory striatum does not produce movements outside the trained discrimination task: rather it results in a choice bias consistent with a role encoding task-relevant information about the value of auditory stimuli (Guo et al., 2018).

Ventral striatum is considered an important link between limbic systems encoding reward and aversion and motor circuits (Mogenson et al., 1980; Carlezon and Thomas, 2009). It receives afferents from ventral areas of mPFC, the basal amygdaloid complex, hippocampus, and limbic areas of cortex and thalamus and sends efferents to ventral pallidum and substantia nigra (Groenewegen et al., 1996). Activation of ventral striatum reduces, while inhibition increases consumption of palatable foods, consistent with a role for ventral striatum in control of feeding based on emotional salience or hedonic value of food (Kelley et al., 2005; Krause et al., 2010). Electrophysiological studies have shown that ventral striatal neurons respond to delivery of action outcomes, stimuli predicting them, and probability and vigor of actions associated with outcome delivery and consumption (Roitman et al., 2005; Taha and Fields, 2005; Morrison et al., 2017; Wright and Wesson, 2021). Ventral pallidal neurons also respond to action outcomes, predictive stimuli, and vigor of outcome-related actions, neuronal responses that are earlier and more robust than ventral striatum (Richard et al., 2016; Ottenheimer et al., 2018; Lederman et al., 2021). This suggests that ventral pallidal responses reflect sensory and motivational signals from other mesocorticolimbic sources. A subset of ventral pallidal neurons encode reward prediction errors indicative of a role computing feedback signals influencing adaptive reward seeking (Ottenheimer et al., 2020).

**FIGURE 8:**
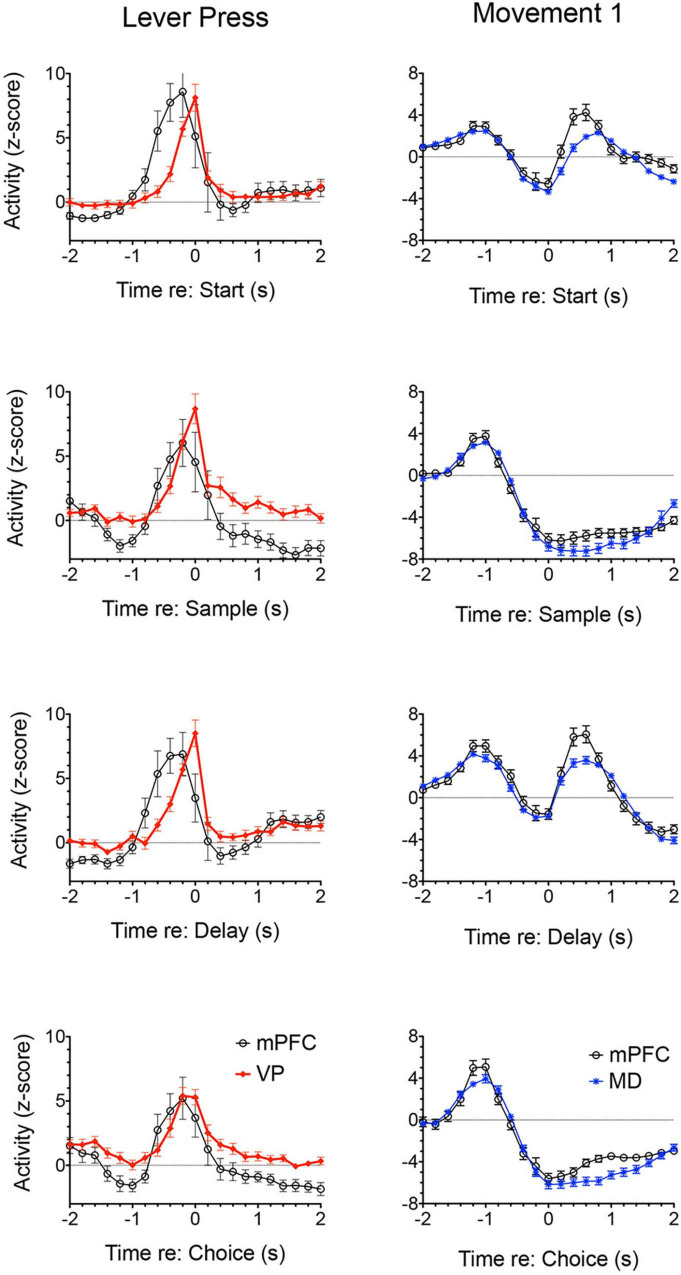
Normalized population histograms comparing responses of neurons with elevated activity during each lever press in mPFC (*n* = 28) and ventral pallidum (VP; *n* = 22) and neurons with elevated activity during movements toward each lever press for mPFC (*n* = 97) and mediodorsal thalamus (MD; *n* = 91). Results are shown for all neuronal responses recorded for each response type with a minimum of 40 trials completed in a 60 m session. These were averaged for individual neurons and normalized so that each response recorded contributed equally to the population function. Error bars represent standard error of the mean.

Lesions or inactivation of ventral pallidum produce delay-dependent impairment of RT and accuracy of DMTP, comparable to effects of mPFC, central thalamic, and ventral striatal lesions (Figure 6; Zhang et al., 2005). Cross-inactivation studies indicate that this impairment is related to disruption of ventral striato-pallido-thalamic pathways (Porter et al., 2001). We recently recorded neural activity in ventral pallidum in rats performing the dynamic DNMTP task using the same methods in earlier studies of mPFC and MD (Onos et al., 2016; Miller et al., 2017; Krell et al., 2019). We found a predominance of neurons exhibiting excitation during delivery of reinforcement with smaller numbers excited during lever press responses and movement toward reinforcement and still fewer with suppressed activity during reinforcement (Figure 7). The timing of these reinforcement-related responses is consistent with populations of neurons exhibiting similar responses in mPFC and MD thalamus (Figure 3). Lever press responses were earlier in mPFC than in ventral pallidum (VP; Figure 8), suggesting a role for mPFC initiating these responses and for VP providing a feedback signal. Interestingly, no neurons were observed with reinforcement anticipation, preparatory, or delay-related responses. These results are consistent with results for simpler discrimination tasks (Richard et al., 2016; Ottenheimer et al., 2018, 2020; Lederman et al., 2021) that VP provides feedback about action outcomes and affects the vigor of outcome-related responses but is not related to prospective processes anticipating incentive value of action outcomes, selecting motor goals, or preparing conditions to execute action sequences.

## Multiple Neural Networks Interact to Support Adaptive Goal-Directed Behavior

Medial prefrontal cortex is organized as a hub for multiple large-scale neural networks that give rise to prospective, concurrent, and retrospective processes that support adaptive goal-directed behavior (Figure 2). Electrophysiological results suggest that prospective processes that anticipate action outcomes, represent goal information during delays preceding actions, and prepare forthcoming motor actions originate in cortex. We compared neuronal activity in rats performing the same DNMTP task and found mPFC neurons with preparatory and delay-related responses not observed in MD as well as earlier and more robust responses anticipating reinforcement than in MD (Figures 3, 7). None of these responses were observed in ventral pallidum. Other studies have described populations of mPFC neurons with activity related to reward anticipation, prospective memory delays, and action preparation in primates and rodents performing other behavioral tasks (see above). Both electrophysiology and neuroimaging studies indicate that activity related to motor action preparation originates in motor, prefrontal, parietal, and sensory areas of cortex (Churchland et al., 2010; Fried et al., 2011; Di Russo et al., 2017). Delay-period activity has been observed in MD for monkeys performing oculomotor delayed response tasks (Fuster and Alexander, 1971; Watanabe and Funahashi, 2004). Population vector analyses indicate that oculomotor delay-period activity in both dorsolateral PFC and MD shifts from encoding antecedent sensory information to prospective motor signals during the memory delay and that this transition occurs earlier in MD (Funahashi, 2013). These results have been interpreted as evidence that reciprocal interactions between primate MD and dorsolateral PFC underlie this transformation of antecedent sensory to prospective motor information and its representation in working memory. Given the lack of a clear homolog of primate dorsolateral PFC in rodents (Vogt et al., 2013; Vogt and Paxinos, 2014) and questions about what information is represented by delay-related activity in rodent mPFC (Euston et al., 2012) it is not clear if these results are relevant to MD function in the rat. Bolkan et al. (2017) have shown that optogenetic inhibition of MD terminals in mPFC in the rat suppresses delay-period activity in mPFC and impairs behavioral performance of a T-maze DNMTP task. Recordings of neuronal activity in ventral pallidum provide an interesting comparison for mPFC. Here, neurons exhibit a subset of responses observed in mPFC (Figure 7), representing information about reward delivery, stimuli that signal outcomes, outcome-related responses, and reward prediction errors and not outcome anticipation, prospective motor goals, or movement preparation (see above). This suggests a role for ventral pallidum providing feedback or a learning signal distinct from prospective functions associated with mPFC (Smith et al., 2009; Ottenheimer et al., 2018, 2020).

Anatomical evidence indicates that mPFC is organized along a dorsal to ventral gradient, with more dorsal areas closely linked to sensory and motor cortices and ventral areas of mPFC receive more prominent connections with amygdala and limbic areas of cortex, including hippocampal and parahippocampal areas (Figure 2; Heidbreder and Groenewegen, 2003; Hoover and Vertes, 2007). Lesion and inactivation studies have described parallel patterns of behavioral impairment that reflect these anatomical specializations (see Dalley et al., 2004; Chudasama, 2011; Kesner and Churchwell, 2011). Hardung et al. (2017) have extended this to adjacent areas of orbital frontal cortex, describing a gradient from proactive motor control in PL to reactive control in ventral and lateral orbital subareas. Electrophysiological studies have not revealed sudden transitions in behavioral event-related response properties of neurons as electrodes are driven ventrally in awake, behaving rats. Systematic mapping analyses have revealed significant trends for more neurons to respond during motor preparation, lever press actions, and non-specific movements in dorsal mPFC and more in ventral mPFC related to reinforcement anticipation, memory delays, and movement toward rewards in rats performing a DNMTP task (Francoeur and Mair, 2018).

Medial prefrontal cortex has reciprocal connections with multiple central thalamic nuclei that have distinct effects on adaptive goal-directed responding (Figure 2). This includes spatial memory functions that are spared by mPFC lesions, but impaired by treatments disrupting anterior, ventral midline, and rostral intralaminar thalamic nuclei as well as dorsomedial striatum (Figure 4; see above). Lesions of anterior thalamic nuclei have delay-dependent effects on varying choice radial maze DNMTP consistent with the effects of hippocampal lesions and indicative of rapid decay of working memory (Figure 4; Mair et al., 1998, 2003). By contrast treatments disrupting intralaminar or ventral midline thalamic nuclei produce delay-independent deficits for this task, consistent with the effects of dorsomedial striatal lesions (Figure 4; Mair et al., 1998, 2002; Hembrook et al., 2012): impairments not observed with intralaminar lesions that spare anterior regions of the intralaminar nuclei (Bailey and Mair, 2005). These results are consistent with evidence that the anterior thalamic nuclei specifically affect hippocampal-dependent spatial memory (Aggleton and Nelson, 2015; O’Mara and Aggleton, 2019) while midline and intralaminar nuclei affect processing of information by medial spinal neurons in striatum (Doig et al., 2010). Although mPFC has substantial projections that presumably influence the activity of dorsomedial striatum and the anterior, ventral midline, and rostral intralaminar nuclei, mPFC lesions spare varying choice DNMTP (Figure 4). Thus mPFC appears to have a modulatory effect on the activity of these areas but is not a critical part of circuits required to perform this task.

Mediodorsal is the main source of focal thalamic projections to middle layers of mPFC and accordingly is often emphasized in treatments analyzing the role of thalamus in mPFC function. Recent studies have elucidated a role for MD regulating signal processing properties of mPFC neurons and stressed its importance for rapid trial-by-trial learning and complex decision making (Mitchell and Chakraborty, 2013; Mitchell, 2015; Mukherjee et al., 2021; see above). The rostral intralaminar nuclei are organized to control cortico-cortical and corticostriatal interactions (Groenewegen and Berendse, 1994; Saalmann, 2014; Phillips et al., 2021). Lesions of these nuclei have more widespread effects on behavior than MD lesions, more closely resembling effects of mPFC and striatal lesions (Mair et al., 2021; Figures 4–6; see above). Reversible inactivation of central thalamus, with treatments producing behavioral impairments comparable to intralaminar or mPFC lesions (Newman and Mair, 2007; Mair and Hembrook, 2008) has a broad effect diminishing the expression of neuronal responses related to actions and outcomes in mPFC for rats performing a DNMTP task (Francoeur et al., 2019). Individually central thalamic nuclei have distinct effects on adaptive goal-directed behavior. Collectively they can account for deficits produced by mPFC lesions and additionally include effects on spatial memory associated with hippocampal, but not prefrontal, pathology.

Dorsal and ventral areas of striatum are comprised of distinct subdomains defined by open loop projections from functionally specific areas of cerebral cortex and the limbic system, closed loop projections from subregions of prefrontal cortex, and thalamostriatal projections primarily from midline and intralaminar nuclei. Both electrophysiological and behavioral findings (reviewed above) are consistent with the hypothesized distinction between ventral striatum processing reward signals and facilitating the motivational control of performance and dorsal striatum forming associations between sensory, motor, and limbic inputs to guide action selection and support the acquisition of goal-directed actions (Hart et al., 2014; Koechlin, 2014). Large-scale mapping studies, primarily in mice, have revealed parallel circuits that interconnect nodes in striatum, pallidum/substantia nigra, thalamus, and mPFC that appear organized to mediate distinct aspects of adaptive behavior (Alexander et al., 1986; Lee et al., 2020; Foster et al., 2021). Like central thalamus, lesions in discrete areas of striatum produce deficits comparable to the effects of lesions in anatomically related areas of mPFC (see above). Thus, at the level of broad channels of information flow in dorsolateral, dorsomedial, and ventral striatum there is very good evidence that pathways involving the basal ganglia and central thalamus are critical for mediating specific aspects of mPFC function. It should be emphasized, however, that striatum does not represent a simple topographic map of mPFC function. Dense projections from subdomains of mPFC overlap considerably in striatum and this overlap is undoubtedly increased by diffuse areas of corticostriatal projections that surround these dense projections (Mailly et al., 2013; Haber, 2016; Heilbronner et al., 2016; Hintiryan et al., 2016). Large-scale mapping studies have revealed complex patterns of convergence, divergence, and reconfiguration of corticostriatal projections: findings that indicate considerable integration in striatum of information originating from different cortical networks (Hintiryan et al., 2016; Hunnicutt et al., 2016).

Thalamus serves as an important integrative center for cortico-basal ganglia networks as nodes connecting basal ganglia to cortex and as the source of thalamostriatal projections that terminate on medium spiny neurons in striatum (Groenewegen and Berendse, 1994; Haber and Calzavara, 2009; Doig et al., 2010; Foster et al., 2021). MD is an important target of ventral striatum via projections to VP and VP projections directly and indirectly (via the thalamic reticular nucleus) to MD (O’Donnell et al., 1997). A further level of complexity is added by thalamostriatal projections from midline and intralaminar nuclei that appear organized to control the integration of cortical and limbic inputs by medium spiny neurons in striatum (Doig et al., 2010). The importance of these thalamostriatal projections has been revealed by behavioral evidence that intralaminar and striatal lesions produce comparable deficits. Thus, intralaminar lesions produce delay-independent impairment of RT and accuracy for operant DMTP, consistent with lesions of ventral and dorsomedial striatum (Figure 6; Burk and Mair, 1998, 2001a); delay-independent impairment of varying- and recurring-choice radial maze DNMTP consistent with lesions of ventral and dorsomedial striatum (Figure 4; Mair et al., 1998, 2002; Bailey and Mair, 2004); and VSRT deficits consistent with dorsolateral striatal lesions (Figure 4; Burk and Mair, 2001b; Bailey et al., 2002; Mair et al., 2002). Similarly, Kato et al. (2011, 2018) used selective immunotoxic methods to demonstrate distinct roles for thalamostriatal projections originating in the parafascicular and central lateral intralaminar nuclei on the acquisition and performance of a visual discrimination task, consistent with functions mediated by dorsal striatal regions innervated by these nuclei.

## Conclusion

(1) Medial prefrontal cortex (mPFC) interacts with the basal ganglia and thalamus to support functions that allow organisms to control actions intended to obtain desired outcomes (or goals). These include prospective anticipation of potential action outcomes, selection and maintenance of motor goals, and motor preparation; concurrent control and monitoring of ongoing actions; and retrospective updating of information and strategies to guide future behavior.
(2) Medial prefrontal cortex is organized with dorsal regions prominently connected with sensory and motor cortices controlling motor planning, prospective decision making, motor response memory, and flexible responding based on trial-specific sensory cues. Ventral areas have more prominent connections with hippocampus, amygdala, and limbic areas of cortex and are important for anticipating action outcomes, fear learning and extinction, and systems memory consolidation.
(3) Electrophysiological recordings during DNMTP reveal neurons with responses related to actions and outcomes distributed throughout mPFC. Although there is no sudden transition apparent when electrodes are driven ventrally through mPFC, there are significant trends for neurons with responses related to motor preparation, lever press actions, and movements between levers to be distributed in dorsal mPFC and neurons with responses related to reward anticipation, movements toward reinforcement, and memory delay to be more frequent in ventral mPFC. Inactivation of central thalamus affects the expression of action- and outcome-related responses in mPFC. The inclusion of choice responses during DNMTP trials engages mPFC neurons to respond to task-relevant information.
(4) Lesion studies indicate that dorsal striatum is important for associative and sensorimotor control and ventral striatum for reward and motivational control of goal-directed behaviors. Deficits produced by striatal lesions parallel the effects of lesions damaging anatomically related areas of mPFC. Large scale mapping studies have described extensive reconfiguration and integration of corticostriatal projections into striatal subnetworks that provide a structural basis for information processing and functional heterogeneity within striatum.
(5) Multiple thalamic nuclei have afferent and efferent connections with mPFC that appear organized to control different aspects of goal-directed responding. The mediodorsal nucleus (MD) is the main source of focal thalamic input to middle layers of mPFC. Recent evidence indicates that MD amplifies and sustains activity in mPFC neurons that encodes information about actions and outcomes important for rapid trial-by-trial learning, complex decision making, and working memory. The intralaminar nuclei have thalamostriatal and thalamocortical projections that control transmission of information in cortico-basal ganglia networks. Behavioral studies have confirmed that intralaminar lesions have broad effects on functions that depend on mPFC and striatum. The ventral medial nucleus has prominent connections with dorsal regions of mPFC and adjacent sensorimotor cortex that support integrative motor responses. The ventral midline reuniens and rhomboid nuclei provide a critical link between mPFC and hippocampus and play a critical role in spatial and contextual memories and systems memory consolidation. The interoanteromedial (IAM) and anterior medial (AM) nuclei are nodes in pathways linking mPFC with hippocampus important for allocentric spatial learning and memory. While lesion studies indicate that mPFC lesions do not impair allocentric spatial memory, reciprocal connections with AM and IAM provide a link between mPFC and hippocampal-related systems that mediates this function.
(6) Comparisons of neuronal activity in mPFC, MD thalamus, and ventral pallidum (VP) reveal important similarities and differences for information represented in these areas in rats performing a dynamic DNMTP task. Neurons in mPFC exhibit responses related to motor preparation, reward anticipation, and prospective memory delays not observed in VP. Responses related to motor preparation and prospective memory delays are also not observed in MD. Although reward anticipation responses are observed in MD, these are delayed and less robust. This suggests that mPFC exerts top-down control of prospective processes anticipating action outcomes, selecting motor goals, and preparing to execute action sequences. VP, by contrast, is dominated by neurons with responses related to reward delivery and reward-related actions consistent with evidence that VP provides feedback about action outcomes and affects the vigor of outcome-related responses.

## Author Contributions

RM was primarily responsible for writing the article. MF, EK, and BG contributed to the writing and discussion. All authors contributed to the article and approved the submitted version.

## Conflict of Interest

The authors declare that the research was conducted in the absence of any commercial or financial relationships that could be construed as a potential conflict of interest.

## Publisher’s Note

All claims expressed in this article are solely those of the authors and do not necessarily represent those of their affiliated organizations, or those of the publisher, the editors and the reviewers. Any product that may be evaluated in this article, or claim that may be made by its manufacturer, is not guaranteed or endorsed by the publisher.
